# Preclinical Studies of Granulysin-Based Anti-MUC1-Tn Immunotoxins as a New Antitumoral Treatment

**DOI:** 10.3390/biomedicines10061223

**Published:** 2022-05-24

**Authors:** Patricia Guerrero-Ochoa, Raquel Ibáñez-Pérez, Germán Berbegal-Pinilla, Diederich Aguilar, Isabel Marzo, Francisco Corzana, Martha Minjárez-Sáenz, Javier Macías-León, Blanca Conde, Javier Raso, Ramón Hurtado-Guerrero, Alberto Anel

**Affiliations:** 1 Apoptosis, Immunity and Cancer Group, Aragón Health Research Institute (IIS-Aragón), University of Zaragoza, 50009 Zaragoza, Spain; docenteashlaboratorio@gmail.com (P.G.-O.); raquelip@unizar.es (R.I.-P.); germanberbegalpinilla@gmail.com (G.B.-P.); imarzo@unizar.es (I.M.); bconde@unizar.es (B.C.); 2 Department of Food Technology, Facultad de Veterinaria, Instituto Agroalimentario de Aragón-IA2, Universidad de Zaragoza-CITA, 50013 Zaragoza, Spain; diederichaguilarmac@uadec.edu.mx (D.A.); jraso@unizar.es (J.R.); 3 Research Center for Chemical Synthesis, Department of Chemistry, University of La Rioja, 26006 Logroño, Spain; francisco.corzana@unirioja.es; 4 Biocomputation and Physics of Complex Systems Institute (BIFI), University of Zaragoza, 50018 Zaragoza, Spain; m_minjarez@live.com.mx (M.M.-S.); jvmacleo@gmail.com (J.M.-L.); rhurtado@bifi.es (R.H.-G.); 5 ARAID Foundation, University of Zaragoza, 50018 Zaragoza, Spain; 6 Copenhagen Center for Glycomics, Department of Cellular and Molecular Medicine, University of Copenhagen, 2200 Copenhagen, Denmark; 7 Laboratorio de Microscopías Avanzada (LMA), University of Zaragoza, 50018 Zaragoza, Spain

**Keywords:** immunotoxins, granulysin, Tn antigen, MUC1

## Abstract

Two granulysin (GRNLY) based immunotoxins were generated, one containing the scFv of the SM3 mAb (SM3GRNLY) and the other the scFv of the AR20.5 mAb (AR20.5GRNLY). These mAb recognize different amino acid sequences of aberrantly O-glycosylated MUC1, also known as the Tn antigen, expressed in a variety of tumor cell types. We first demonstrated the affinity of these immunotoxins for their antigen using surface plasmon resonance for the purified antigen and flow cytometry for the antigen expressed on the surface of living tumor cells. The induction of cell death of tumor cell lines of different origin positive for Tn antigen expression was stronger in the cases of the immunotoxins than that induced by GRNLY alone. The mechanism of cell death induced by the immunotoxins was studied, showing that the apoptotic component demonstrated previously for GRNLY was also present, but that cell death induced by the immunotoxins included also necroptotic and necrotic components. Finally, we demonstrated the in vivo tumor targeting by the immunotoxins after systemic injection using a xenograft model of the human pancreatic adenocarcinoma CAPAN-2 in athymic mice. While GRNLY alone did not have a therapeutic effect, SM3GRNLY and AR20.5GRNLY reduced tumor volume by 42 and 60%, respectively, compared with untreated tumor-bearing mice, although the results were not statistically significant in the case of AR20.5GRNLY. Histological studies of tumors obtained from treated mice demonstrated reduced cellularity, nuclear morphology compatible with apoptosis induction and active caspase-3 detection by immunohistochemistry. Overall, our results exemplify that these immunotoxins are potential drugs to treat Tn-expressing cancers.

## 1. Introduction

The Tn antigen is mostly known as an aberrant hypoglycosylation of the MUC-1 membrane protein. This consists of an N-acetylgalactosamine (GalNAc) O-linked glycosylation and occurs at serine (Ser) or threonine (Thr) residues of glycoproteins [1]. Following the formation of the Tn antigen, the GalNAc residue can be further modified by distinct glycosyltransferases and construct different glycan structures. MUC-1 was the first transmembrane mucin to be identified and structurally characterized [2]. The aberrantly O-glycosilation of MUC 1 occurs in most epithelial cancers [3], and has been classified by the National Cancer Institute as one of the most promising tumor antigens to be targeted by new treatments [4].

It has been clearly demonstrated that the expression mechanisms of the Tn antigen result from genetic changes that lead to decreased expression of Cosmc and/or T synthase [5]. However, there are other possible factors that could influence the glycosylation and expression pathways of the Tn antigen, such as UDP-Gal or its transporter [2].

In the mid-1990s, 56 anti-MUC1 monoclonal antibodies were compared and their epitopes mapped. Among others, the anti-MUC1 SM3 and AR20.5 mAb, whose reactivity is altered due to glycosylation, were evaluated [6]. They recognize a six amino acid sequence of MUC1 [7,8]. In normal cells, the heavy O-glycosylation of the VNTR sequence of 20–21 amino acids masks the peptide core and protects it from cleavage by proteolytic enzymes, and also prevents it from undergoing clathrin-mediated endocytosis [1]. MUC1 and some growth factors are confined to the apical and basolateral surface of normal epithelium. The aberrant hypoglycosylation of MUC1 correlates with the loss of apicobasal polarity, so that it is localized throughout the cell surface [1].

Early reports showed the antitumor capacity of SM3 in different experimental settings [9,10] and, afterwards, it has been used to generate effective anti-tumor CAR T cells [11,12]. The initial non-humanized version of AR20.5 was used in a clinical assay upon the commercial name of Brevarex^®^, but the xenogenic response mostly blocked the effects of the treatment [13]. A recent preclinical study using a humanized version of AR20.5 in combination with poly:ICLC and with anti-PD-L1 mAb gave excellent results against pancreatic tumors [14].

Recently, the immunotoxin moxetumomab pasudotox, an anti-CD22 scFv fused with the *Pseudomonas* Exotoxin A PE38 has been approved for the treatment of hairy cell leukaemia [15]. Another PE38-based immunotoxin, oportuzumab monatox, which includes an scFv directed against EpCAM was in review as of August 2021 for bladder cancer treatment [16]. On the othe hand, our research group has studied the antitumor capacity of recombinant 9 kDa granulysin (GRNLY) for more than two decades [17,18,19,20]. Previous studies showed the in vivo tumor targeting of MFE23GRNLY, an immunotoxin engineered against the carcinoembryonic antigen (CEA), after systemic injection [21]. This therapy would be directed to tumors with high expression of CEA, mostly colorectal or gastric tumors [16]. Since the expression of the Tn antigen is observed in a much wider variety of tumor types, targeting this antigen would expand the possible therapeutic applications of granulysin-based immunotoxins. In order to optimize the antitumor capacity of Tn antigen-targeted therapies, we have designed two immunotoxins combining SM3 and AR20.5 with GRNLY.

The standard protocol to obtain recombinant proteins on *P. pastoris* (*Komagataella phaffii*), previously used by our group to produce MFE23GRNLY [21], did not permit the obtainment of enough of the new anti-Tn immunotoxins to face in vivo experiments. For this reason, various steps of the production and purification process were modified in search of an optimized protocol. In addition, we used a non-thermic technology, yeast electroporation by pulsed electric fields (PEF), to obtain the intracellular protein and increase the yield of production [22].

## 2. Materials and Methods

### 2.1. Bacterial Strains, Plasmids, and Culture Conditions

Synthetic genes encoding 6xHis-tagged 9 kDa granulysin, SM3GRNLY or AR20.5GRNLY were synthesized by Geneart GmbH (Thermo Fisher Scientific, Regensburg, Germany) and subcloned as ClaI/XbaI or NruI/XbaI into pCR3.1-NC145 resulting in pCR3.1-GRNLY, pCR3.1-SM3GRNLY, and pCR3.1-AR20.5GRNLY, respectively. The ClaI/XbaI-digested fragments of pCR3.1-GRNLY, pCR3.1-SM3GRNLY, and pCR3.1-AR20.5GRNLY were ligated into the ClaI/XbaIdigested backbone of plasmid pPICZα to obtain pPICZαC-GRNLY and pPICZαA-SM3GRNLY or pPICZαA-AR20.5GRNLY. The *E. coli DH5* strain was grown at 37 °C in Luria-Bertani medium (LB; Oxoid, Basingstoke, UK). *Pichia pastoris* was grown at 30 °C in yeast extract with peptone and dextrose (YPD) broth (Formedium) for routine maintenance and in buffered glycerol-complex medium (BMGY) (1% yeast extract, 2% peptone, 1.34% yeast nitrogen base (YNB) 1% glycerol, 400 g/L biotin, and 0.1 M potassium phosphate, pH 6.0) for expansion and big-scale production, followed by cultured at 18 °C in buffered methanol-complex medium (BMMY) (1% yeast extract, 2% peptone, 1.34% YNB, 1% methanol, 400 g/L biotin, and 0.1 M potassium phosphate, pH 6.0) for induction of the recombinant protein. The synthetic gene encoding 6xHis-tagged 9 kDa granulysin was synthesized and inserted in the *pPICZαA* plasmid as indicated in [21]. The synthetic gene coding for 6xHis-tagged SM3GRNLY was synthesized and inserted in the pPICZ A plasmid by Genscript (Leiden, The Netherlands). Both plasmids were amplificated in *E. coli* and isolated by Nucleo SpinPlasmid EasyPure (Macherey-Nagel). Plasmids were linearized with *SacI* (Takara) and purified by Ilustra GFX PCR DNA and Gel Band Purification kit (GE Healthcare). The transformation of *P. pastoris* and the selection of transfected colonies was performed by the methods described in [21].

### 2.2. Expression and Purification of Extracellular Recombinant GRNLY and Immunotoxins in Pichia Pastoris

The pre-inoculum of *P. pastoris* cell strains *X33* for SM3GRNLY and AR20.5GRNLY and *SMD1168* for GRNLY, was cultured in 100 mL YPD medium overnight at 30 °C for activation; the cells were then added to 1000 mL of BMGY medium, incubated overnight at 30 °C for growth and finally in BMMY medium at 18 °C for induction. All these culture steps were performed in a thermostated shaking incubator at 250 rpm as shaking intensity. The culture was fed with 1% methanol every 24 h for 2 or 3 days; then the culture was centrifuged 30 min and the supernatant was incubated with Ni^2+^ affinity chromatography (Ni-NTA-agarose overnight-Invitrogen) and eluted with buffer Imidazol 250 mM in PBS. The eluate was concentrated and its buffer was changed to PBS using Amicon filters (MerckMillipore, Burlington, MA, USA).

### 2.3. Expression and Purification of Intracellular Recombinant Immunotoxins from Pichia Pastoris by Pulsed Electric Field Technology

The PEF equipment used in this work was the commercial model EPULSUS-PM1-10 (Energy Pulse System, Lisbon, Portugal). *P. pastoris* cells were resuspended in McIlvaine buffer (pH 7.0 and 1.50 mS/cm) at a concentration of around 108 cells/mL and PEF-treated in continuous flow (5.0 L/h) in parallel electrode chamber of 3.0 cm length, 0.50 cm width and a gap of 0.40 cm. The calculated mean residence time in the treatment chamber was 0.40 s. A heat exchanger consisting of a coil submerged in a thermostatic batch was used to set the initial temperature of the yeast before the treatment at 10 °C. The temperature of the yeast suspension after the PEF treatment chamber never exceeded 30 °C. *P. pastoris* cells were treated at electric field strength between 8 and 20 kV/cm for treatment times between 150 and 180 μs. After the treatments, serial decimal dilutions were poured plated in potato dextrose agar in order to monitor *P. pastoris* cell inactivation. The number of viable cells was expressed in colony-forming units (CFU), corresponding to the number of colonies counted after 48 h of incubation at 25 °C. Inactivation data were expressed as the ratio between the initial number of survivors (No) and the number of survivors after different treatment times (Nt). Release of protein from untreated and PEF-treated (12, 16 and 20 kV/cm for 15 to 180 μs) cells of *P. pastoris* was monitored after 180 min of incubation at 20 °C in a McIlvaine buffer solution of pH 7 and 1 mS/cm of conductivity. Quantitative analysis of released proteins was conducted by the microplate procedure of Pierce BCA Protein Assay Kit (Thermo Scientific, Rockford, IL, USA) [22].

### 2.4. Surface Plasmon Resonance

SPR experiments were performed with a Biacore X-100 apparatus (Biacore, GE, Uppsala, Sweden) in HBS-EP buffer at pH 7.5 (Hepes 10 mM, NaCl 150 mM, EDTA 3 mM, with 2% DMSO and 0.05% Tween X100 as the running buffer at 25 °C. The SM3GRNLY and AR20.5GRNLY immunotoxins were immobilized on a CM5 sensor chip (Biacore, GE) following standard amine coupling method. Briefly, the carboxymethyl dextran surface of the flow cell 2 was activated with a 7-min injection of a 1:1 ratio of aqueous 0.4 M 1-ethyl-3-(3-dimethylaminopropyl) carbodiimide (EDC) and 0.1 M sulfo-N-hydroxysuccinimide. Then, the SM3GRNLY and AR20.5GRNLY immunotoxins was coupled to the surface during a 7-min injection using several dilutions in 10 mM sodium acetate, pH 4.0. The unreacted active esters on the surface were quenched by a 7-min injection of aqueous 0.1 M ethanolamine-HCl (pH 8.0). The levels of immobilization were 5700 resonance units (RU) for SM3GRNLY and 6800 RU for AR20.5GRNLY. Flow cell 1 treated as a flow cell 2 (amine coupling procedure) without protein was used as a reference. Prior to use, 50 mM stock solutions of the peptide ligand MUC21T were diluted to the final concentration in the running buffer. The MUC21T peptide is a tandem-repeat sequence of MUC1, where the Thr marked with (*) is glycosylated with alpha-GalNAc, with the following sequence: AHGVTSAPDT*RPAPGSTAPPA. An Ala residue (bold) was added to the N-terminal extreme to facilitate binding to the Au in the sensor. Typically, a series of different compounds was injected onto the sensor chip a flow rate of 30 μL/min for a period of 1min followed by a dissociation period of 1 min. No regeneration was needed. The concentrations used for affinity measurements were in the range of 0.05–4 mM. Sensogram data were double-referenced using the Biaevaluation X-100 software (Biacore, GE). The experimental data of affinity measurements were fitted to a one site-specific model binding using Prism software. Regarding the affinity of SM3GRNLY or AR20.5GRNLY for its antigen, 390 RUs of MUC21T were immobilized and increasing concentrations of SM3GRNLY or AR20.5GRNLY from 2.5 nM to 1 µM were tested.

### 2.5. Cell Culture

The acute lymphoblastic leukemia Jurkat and multiple myeloma H929 cells, obtained from the ATCC, were cultured in RPMI 1640 medium supplemented with 5% FBS (Pan Biotech, Aidenbach, Germany) at 37 °C and 5% CO_2_ using standard procedures. Derived Jurkat–pLVTHM and JurkatshBak, obtained in our laboratory, were cultured in the same conditions. The pancreatic adenocarcinomas CAPAN-2, PANC-1, MiaPaca2 and derived MiaPaca2 BaxBak double KO, the lung adenocarcinoma A549 and the breast adenocarcinomas MDA-MB-231 and MCF-7 were cultured in DMEM medium (Pan Biotech GmbH) supplemented with 10% FBS (Sigma, St. Louis, MO, USA). In all cases, culture media were supplemented with penicillin/streptomycin (Pan Biotech) and GlutaMAX (Invitrogen, Barcelona, Spain). All cell lines were routinely tested for mycoplasma contamination by PCR.

### 2.6. Binding of Immunotoxins to the Tn Antigen Analyzed by Flow Cytometry

The expression of the MUC1-Tn antigen on the different cell lines used in this study was first analyzed using a commercial SM3 mAb conjugated with FITC (Santa Cruz Biotech sc-53381; Heidelberg, Germany). To analyze binding to the Tn antigen on the surface of living cells, 10^5^ cells per well were placed in a 96-well round bottom plate. First, cells were incubated with or without SM3GRNLY, iSM3GRNLY or AR20.5GRNLY (10 μg/mL) in PBS 5% FBS for 30 min at 4 °C followed by mouse anti-histidine tag antibody (1:200; Genscript, Leiden, The Netherlands) and goat anti-mouse antibody bound to FITC (1:200; Caltag, Barcelona, Spain). After each incubation, cells were washed with 5% FBS in PBS. Binding was determined using a FACScalibur flow cytometer (BD Biosciences, Madrid, Spain) using as controls cells treated in the same way but in the absence of the incubation with the immunotoxin. MUC1-Tn antigen expression on the cell lines used was also confirmed using a commercial SM3 mAb bound to FITC (Santa Cruz Biotech, Heidelberg, Germany)

### 2.7. In Vitro Cytotoxicity Assays

50 μL aliquots of 1 × 10^6^/mL cell suspensions in complete medium were seeded per well in 96 well plates and GRNLY or the immunotoxins were added at the indicated concentrations. In control wells, the same volume of PBS was added. Cells were then incubated for 24 h at 37 °C and cell death was analyzed by determination of phosphatidyl- serine exposure by flow cytometry after incubation with Annexin V-FITC (BD Biosciences, Madrid, Spain) or with 7-AAD in annexin binding buffer (140 mMNaCl, 2.5 mM CaCl_2_, 10 mM Hepes/NaOH, pH 7.4) for 15 min. Annexin V-DY63, produced as described by Logue et al. [23], was used in the experiments involving Jurkat–pLVTHM or JurkatshBak cells. Cell death was estimated using a simultaneous determination of FITC or 7-AAD labelling and cell size by forward scatter (FSC).

### 2.8. In Vivo Assays

Immune-deficient athymic mice, Swiss nu/nu strain, six-week old males (Charles River, Wilmington, MA, USA), were used in this study. Mice experiments were performed according to the European recommendations on animal ethics and the University of Zaragoza Animal Experimentation Ethical Commission previously approved the housing and experimental protocols. Mice were kept under specific standard pathogen-free conditions (average ambient temperature 24 °C, 12/12 h light/dark cycle) with water and food provided ad libitum throughout the study. Tumor growth was analyzed by measuring the tumor daily with a precision caliper. To calculate the tumor volume, the width (A) and length (L) of the tumor were measured, and the following formula was applied: V = L × A^2^/2.

At the end of the experiment (2 days after the last injection) mice were euthanized and the tumors were surgically excised, fixed in 10% buffered formalin and embedded in paraffin. For systemic treatments, 5 × 10^6^ CAPAN-2 tumor cells suspended in Matrigel were injected subcutaneously in nude mice (n = 5 mice per group). When the tumors reached a mean volume of 0.2 cm^3^, mice were treated with intraperitoneal injections of 0.5 nmol/g mouse weight of GRNLY or SM3GRNLY or with 0.2 nmol/g mouse weight of AR20.5GRNLY i.p. every 2 days for 10 times. Mice in the control group received injections of PBS with the same time schedule. Taking into account the molecular weight of the recombinant proteins (11 kDa for GRNLY; 47 kDa for SM3GRNLY; 45 kDa for AR20.5GRNLY), this represents 110 µg of GRNLY, 470 µg of SM3GRNLY and 180 µg of AR20.5GRNLY per injection per mouse considering a mean mouse weight of 20 g.

### 2.9. Histology Analysis

Tissue sections 5 μm thick were deparaffinated, rehydrated and stained by immersing in GILL II Hematoxylin, followed by eosin staining. For the study of apoptotic nuclei, tissue sections were stained with DAPI Fluoromont-G (EMS, Madrid, Spain) for 10 min and detected in a fluorescence microscope (E600/E400, Nikon, Tokyo, Japan) equipped with a digital photography machine (DXM1200F, Nikon). The expression of activated caspase-3 was investigated by immunohistochemistry using a rabbit polyclonal anti-human caspase-3 antibody (Cell Signaling, Barcelona, Spain), which recognizes the active, cleaved caspase-3 form. For antigen retrieval, the sections were boiled in 10 mM citrate buffer, pH 6.0 for 30 min. After blocking with 5% horse serum diluted in PBS for 1 hr at room temperature, sections were incubated at 4 °C in humid chambers with the anti-caspase-3 antibody at 1/150 dilution for 1h followed by ready to use secondary anti-rabbit antibody (Vector Laboratories, Peterborough, UK) for 30 min. As a chromogenic substrate, DAB (Agilent, Madrid, Spain) was used, followed by hematoxylin counterstaining. Appropiate negative control stainings were also performed.

### 2.10. Statistical Analysis

Computer-based statistical analysis was performed using GraphPad Prism 4.0 program (GrandPath Software Inc., Salford, UK). Results are shown as mean SD. Statistical significance was evaluated by using Student *t* test for non-paired variants. Differences were considered significant if *p* < 0.05.

## 3. Results

### 3.1. Design of Recombinant Immunotoxin and Yield of Production

**Figure 1:**
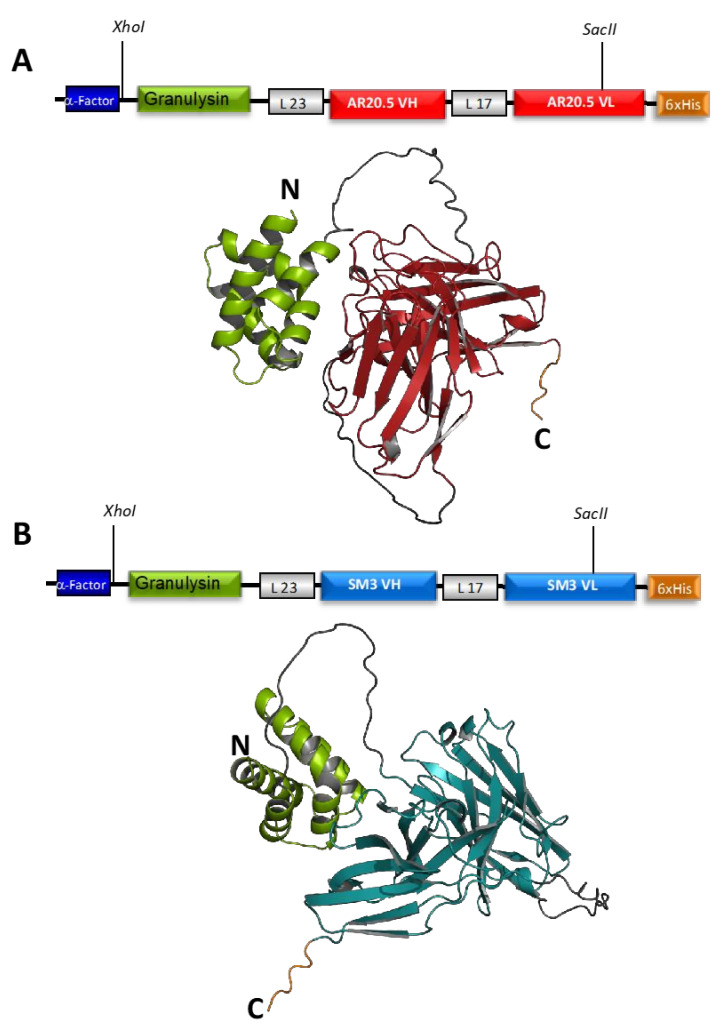
Structural features of immunotoxins. Plasmid constructution and Alpha-Fold structural model of the chimeric protein of the (**A**) immunotoxin AR20.5GRNLY (Granulysin-AR20.5_ScFv-HisTag) and (**B**) SM3GRNLY (Granulysin-SM3_ScFv-HisTag). Sequence of Granulysin portion is show in green, L23 and L17 are show in grey, and fusion protein corresponding to AR20.5 and SM3 are in red and blue, respectively. AlphaFold predictions were made using the AlphaFold.ipynb v1.0 colab notebook as part of the ColabFold framework [24] and visualization of immunotoxins models was performed by PyMOL [25].

The construct coding for the 9 kDa granulysin (GRNLY) gene alone was described in our previous works [21,22]. We designed constructs to encode for the two anti-Tn immunotoxins formed by granulysin bound through a flexible linker of 23 amino acids (formed by Ser and Gly residues) to the anti-Tn scFv SM3 or AR20.5, respectively. A 17 amino acid linker was also introduced between the VL and VH sections of the scFv (Figure 1). All constructs included a tag of six histidines to facilitate detection and purification. Plasmids were amplificated in *Escherichia coli* and isolated. Isolated plasmids were then linearized with SacI and purified. Finally, plasmids were transfected by electroporation in *Pichia pastoris* and the transfected colonies selected, as indicated in Materials and Methods.

As indicated in our previous work [22], the yield of production of recombinant GRNLY was good, around 5 mg/L in the different batches, but the yield of production of SM3GRNLY was lower, around 2 mg/L. Taking into account the difference in molecular weight, this difference in weight yield meant a difference of 10-fold in molar yield. This low yield precluded the development of in vivo experimentation with the immunotoxin, our final objective. We then performed extraction of intracellular SM3GRNLY (iSM3GRNLY) using the PEF technique, and obtained more than 8 mg per liter of yeast medium, four-fold the protein that was secreted to the supernatant. Regarding AR20.5GRNLY, the situation was even more unfavorable, being the yield of the extracellular protein as low as 0.5 mg/L. When applying the PEF technology to obtain the intracellular protein, the yield increases as much as to 12.9 mg/L. All these data are depicted in Supplementary Table S1. However, these iAR20.5GRNLY preparations contained another protein impurity that was not possible to eliminate in the purification process (data not shown). As a consequence, we could only work with the low amounts of extracellular AR20.5GRNLY obtained in the different batches.

### 3.2. Immunotoxin Affinity for Its Antigen In Vitro

**Figure 2:**
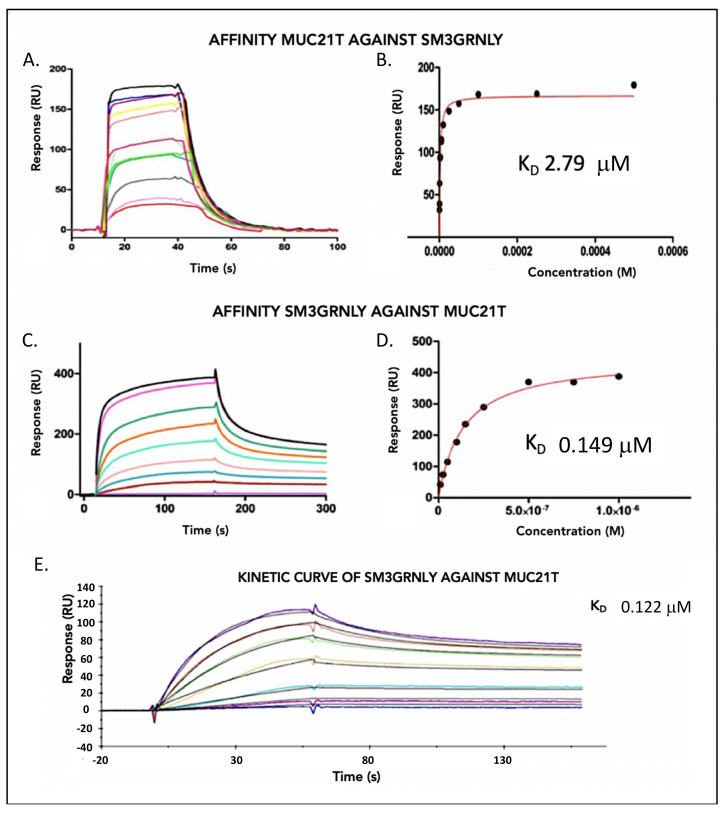
SM3GRNLY-MUC1-Tn SPR Affinity Assays. (**A**,**B**) 5700 RUs of SM3GRNLY were immobilized on the biosensor. The response was measured at different concentrations of the antigen, ranging from 0.05 µM to 500 µM. (**C**,**D**) 390 RUs of MUC1-Tn were immobilized and different concentrations of SM3GRNLY were studied. (**E**) K_D_ calculation using kinetic adjustment. Colored lines correspond to the determinations with the different concentrations used in the titrations.

Using Surface Plasmon Resonance, the affinity of the MUC21T peptide for SM3GRNLY was determined. The MUC21T peptide is a tandem-repeat sequence of MUC1, where the Thr marked with (*) is glycosylated with alpha-GalNAc, with the following sequence: AHGVTSAPDT*RPAPGSTAPPA. The measruments were made by immobilizing 5700 resonance units (RUs) of the immunotoxin on a CM5 sensor chip, using the standard amine protocol. The response measurements were performed with increasing antigen concentrations from 0.5 µM to 500 µM (Figure 2A,B). The K_D_ obtained using this method was of 2.79 µM. Regarding the affinity of SM3GRNLY for its antigen, 390 RUs of MUC21T were immobilized and increasing concentrations of SM3GRNLY from 2.5 nM to 1 µM were tested. The K_D_ obtained was of 0.149 µM (Figure 2C,D). The kinetic adjustment of these last measurements established a K_D_ of 0.122 µM (Figure 2E).

**Figure 3:**
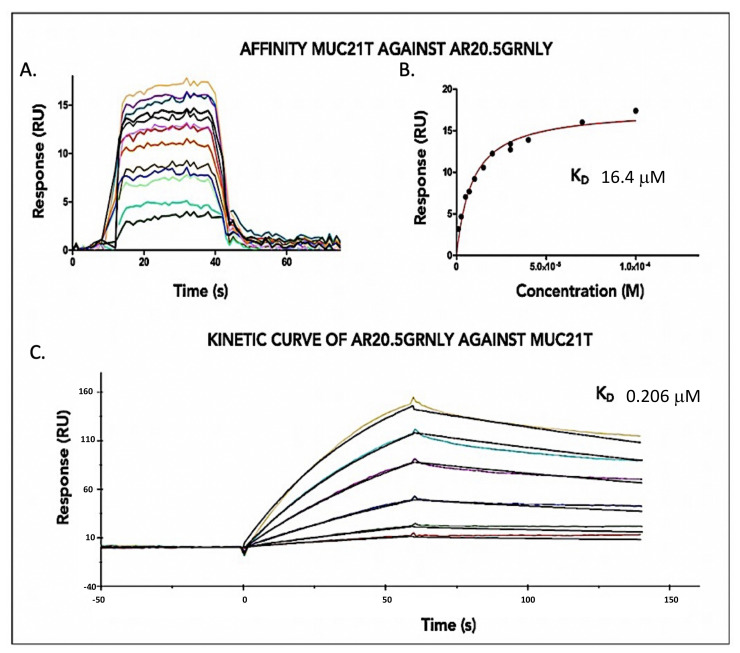
AR20.5GR-MUC1-Tn SPR Affinity Assays. (**A**,**B**) 6800 RUs of AR20.5GRNLY were immobilized on the biosensor, the response was measured with different concentrations of the antigen, varying from 0.07 µM to 1µM. (**C**) Kinetic curve of AR20.5GRNLY when the target was immobilized. Colored lines correspond to the determinations with the different concentrations used in the titrations.

To establish the affinity of MUC21T for AR20.5GRNLY, 6800 RUs of the immunotoxin were immobilized in the biosensor and the response was measured with increasing concentrations of the antigen from 0.07 µM to 1 µM, obtaining a K_D_ of 16.4 µM (Figure 3A,B). Regarding the affinity of AR20.5GRNLY for its antigen, a similar assay as that indicated above for SM3GRNLY was performed. However, the low concentrations of AR20.5GRNLY did not allow performing a reliable K_D_ calculation. The sensogram data were double referenced using the Biaevaluation X-100 software and the experimental data from the affinity measurements were fitted to a site-specific binding pattern using Prism software. The result obtained for this kinetic adjustment was a K_D_ of 0.206 µM (Figure 3C).

The difference in affinity obtained using the two methods reflects the different experimental approach, being the K_D_ obtained by immobilization of the peptide and increasing the immunotoxin concentration more accurately reflecting the physiological situation, in which the immunotoxin in liquid phase should recognize the antigen on a protein expressed on a cell surface. The reported K_D_ for the AR20.5 antibody on Tn was 0.43 µM [8], while for the SM3 antibody it was 0.45 µM [3]. Hence, the affinity of GRNLY-linked immunotoxins for their antigen is in the range of those previously observed for the antibodies themselves. These results demonstrate that the antibody moiety of the immunotoxins conserved their high affinity for the purified MUC1-Tn antigen.

### 3.3. Immunotoxin Recognition of Its Antigen on the Cell Surface

**Figure 4:**
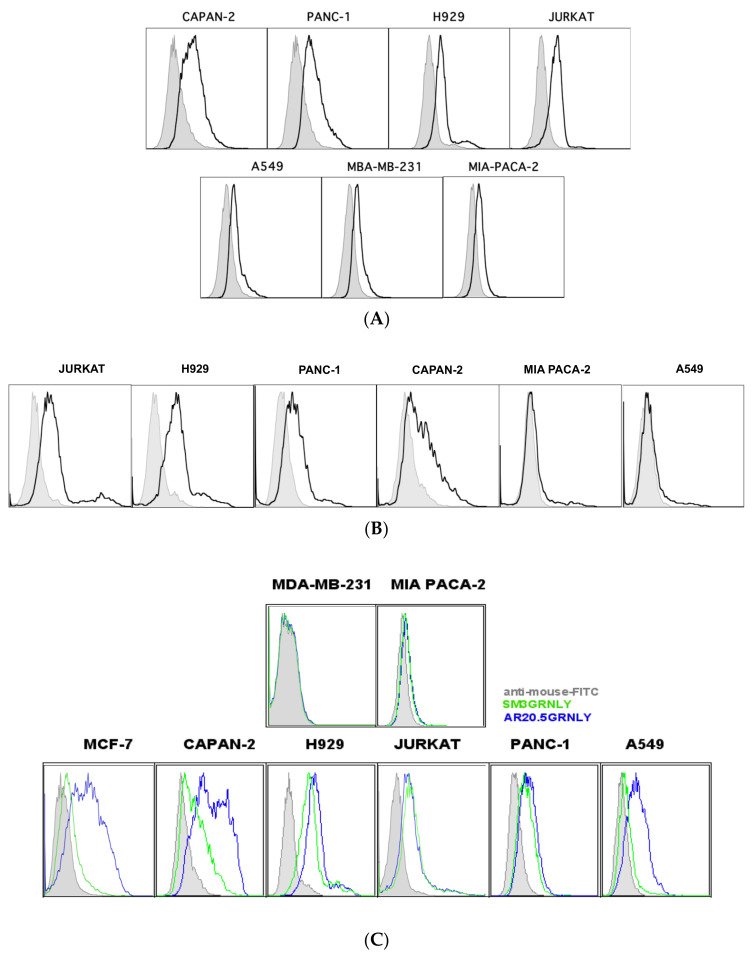
Recognition of the MUC1-Tn antigen on the cell membrane of tumor cell lines. (**A**) expression of the MUC1-Tn antigen was analyzed on different tumor cell lines by flow cytometry using a commercial SM3 mAb conjugated with FITC. Grey histograms correspond to the isotype antibody labeling. (**B**) binding of the SM3GRNLY immunotoxin to tumor cell lines. Shaded histograms correspond to cells incubated with anti-His-tag antibody and FITC-conjugated goat anti-mouse IgG antibody in the absence of the immunotoxin and open histograms correspond to cells sequentially incubated at 4 °C with SM3GRNLY and the mentioned antibodies. (**C**) the same labelings and controls as in B were performed, comparing the binding of the SM3GRNLY (green histograms) and the AR20.5GRNLY immunotoxin (blue histograms). Images are representative of at least 3 different labeling for each cell line.

In our previous study, and using flow cytometry, we demonstrated that both SM3GRNLY and iSM3GRNLY recognized MUC1-Tn expression on the surface of living tumor cells previously reported to be positive for the expression of this antigen, including the pancreatic adenocarcinomas Panc-1 and CAPAN-2, the multiple myeloma NCI-H929 an the acute lymphocytic leukemia Jurkat, with low binding to lung adenocarcinoma A549 [22]. We have corrobarated the expression of this antigen in these cell lines using a commercial SM3 mAb labelled with FITC (Figure 4A), and confirmed the previous observations regarding SM3GRNLY binding to them (Figure 4B).

We have now comparatively tested the binding of AR20.5GRNLY to that of SM3GRNLY in these cell lines and results are depicted in Figure 4C. The labeling with AR20.5GRNLY was similar to that of SM3GRNLY for H929, Jurkat or Panc-1 cells, but it was more intense in the case of CAPAN-2 cells, giving also a positive labelling in the case of the lung adenocarcinoma A549 and the mammary carcinoma MCF7, whose labeling with SM3GRNLY was very low.

The higher increase in labeling, more than four-fold, was observed in the case of the pancreatic adenocarcinoma CAPAN-2. All these labeling were specific, since the Tn-negative cell line MDA-MB-231 [26] was negative for labeling with SM3GRNLY or AR20.5GRNLY, a property shared by the pancreatic adenocarcinoma MiaPaca2 (Figure 4C) [27].

### 3.4. Cytotoxicity Assays on MUC1-Tn^+^ Tumor Cells

**Figure 5:**
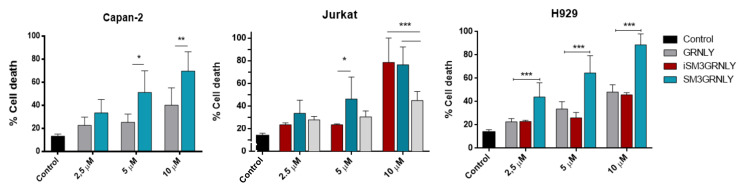
Dose-response cytotoxicity assays of GRNLY, SM3GRNLY and iSM3GRNLY on Capna-2, Jurkat and H929 cells. The cells were incubated with increasing concentrations of the recombinant proteins GRNLY (gray bars), iSM3GRNLY (red bars) or SM3GRNLY (green bars) for 24 h. GRNLY, SM3GRNLY and iSM3GRNLY induced cell death was determined by detection of PS translocation by Annexin-V-FITC labeling combined with size analysis. Results are the mean ± SD of at least three different experiments performed in triplicate. * *p* < 0.05, ** *p* < 0.01, *** *p* < 0.001.

Once the recognition capacity of its antigen was established, the induction of cytotoxicity by immunotoxins was evaluated in comparison with GRNLY. The cytotoxicity of SM3GRNLY and iSM3GRLY was tested in our previous study on MCF7, A549, Capan-2, Panc-1 and H929 cells, correlating with their low (MCF7, A549) or high (Capan-2, Panc-1 and H929) surface binding to the Tn antigen [22]. We have now corroborated these results in the two cell lines that gave a higher binding of the immunotoxin, Capan-2 and H929 (Figure 5), and we have also analyzed their cytotoxicity on Jurkat cells, a cell line with reported high MUC1-Tn expression due to Cosmc mutation [28,29] and with a high binding rate of the immunotoxins (see Figure 4).

As shown in Figure 5, SM3GRNLY resulted significantly more cytotoxic against Jurkat cells than GRNLY alone at 5 µM. This higher cytotoxicity was shared by SM3GRNLY and iSM3GRNLY at 10 µM, demonstrating the increase in cytotoxicity given by antigen recognition by the scFv moiety.

**Figure 6:**
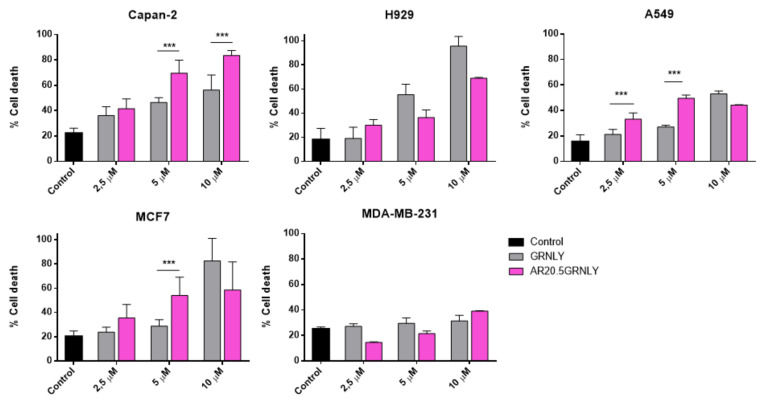
Dose-response cytotoxicity assays of GRNLY and AR20.5GRNLY in CAPAN-2, MCF-7, A549, H929 and MDA-MB-231 cells. Cells were incubated with increasing concentrations of recombinant GRNLY (gray bars), iAR20.5GRNLY (dark gray bars), or AR20.5GRNLY (light gray bars) for 24 h. Cell death was determined by detecting PS exposure by Annexin-V-FITC labeling combined with size analysis (FSC-H). Results are the mean ± SD of at least three different experiments performed in triplicate for each cell line. *** *p* < 0.001.

Regarding AR20.5GRNLY, CAPAN-2 pancreatic adenocarcinoma cells were clearly more sensitive to the immunotoxin than to GRNLY alone (Figure 6). At 5 µM and 10 µM, AR20.5GNLY was significantly more cytotoxic than GRNLY, reaching over 80% cell death at 10 µM. This correlates with the fact that CAPAN-2 is the cell line in which the highest binding of AR20.5GRNLY was found by flow cytometry (see Figure 4). In the case of the multiple myeloma NCI-H929, and although the binding of AR20.5GNLY was also high (Figure 4), the cytotoxicity of AR20.5GRNLY was not higher than that of GRNLY (Figure 6). This could be due to the fact that H929 cells are very sensitive to GRNLY cytotoxicity, as previously described both in vitro [19] and in vivo [17], being the targeting mediated by the scFv moiety unable to increase this cytotoxicity level. In A549 lung adenocarcinoma cells, AR20.5GRNLY resulted significantly more cytotoxic than GRNLY at low concentrations, 2.5 and 5 µM, although this level of cytotoxicity did not increase at 10 µM, arriving at a maximum of 60% of cell death for both GRNLY and AR20.5GRNLY (Figure 6). In the MCF-7 breast adenocarcinoma, AR20.5GRNLY was more cytotoxic than GRNLY at 5 µM, but, as observed in A549 cells, this level of cytotoxicity did not increase at 10 µM (Figure 6). Finally, MDA-MB-231 cells, a breast adenocarcinoma cell line negative for the expression of the MUC1-Tn antigen, were not very sensitive to GRNLY, and this cytotoxicity did not increase when using AR20.5GRNLY, confirming the usefulness of this cell line as a negative control for MUC1-Tn expression.

Of note, neither SM3GRNLY nor AR20.5GRNLY bound to the surface of normal human PBMC (Supplementary Figure S1A). We also confirmed our previous observation on the lack of cytotoxicity of GRNLY on PBMC as compared with leukemic Jurkat cells [19], and demonstrated the absence of cytotoxicity of SM3GRNLY on PBMC, while increasing its cytotoxicity on Jurkat cells (Supplementary Figure S1B).

### 3.5. Mechanism of Cell Death Induced by SM3GRNLY

The GRNLY mechanism of cell death induction was previously shown to be mediated by the mitochondrial apoptotic pathway, although in some cell lines, and especially if caspases were inhibited, a minor necroptotic component could be also observed [19,30]. The mitochondrial apoptotic pathway, through translocation of cytochrome c from mitochondria to the cytosol and formation of the apoptosome, results in the caspase-dependent PS exposure on the external membrane hemileaflet. Hence, analysis of PS exposure by Annexin-V staining and flow cytometry is a convenient method to estimate cell death induction [31].

**Figure 7:**
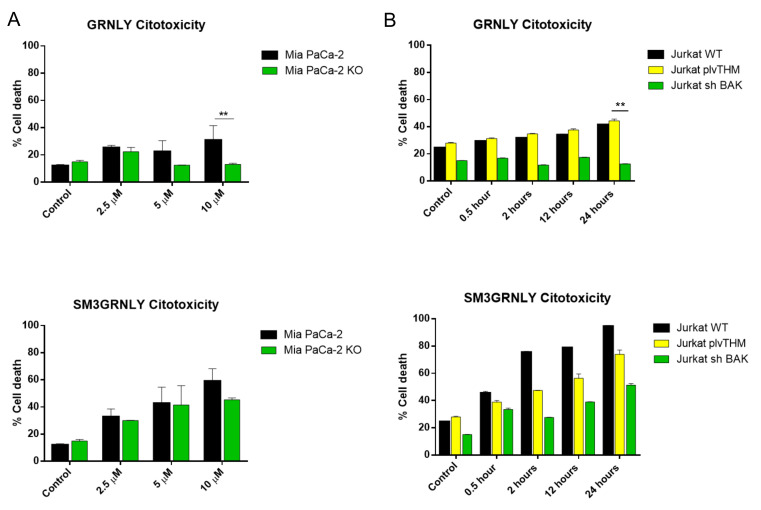
Dose-response cytotoxicity assays of GRNLY and SM3GRNLY on MIA PACA-2 and MIA PACA-2 KO cells (**A**) or on Jurkat, Jurkat-plvTHM and Jurkat-shBak cells (**B**). (**A**) cells were incubated with increasing concentrations of recombinant GRNLY (upper panel) or of SM3GRNLY (lower panel) for 24 h; (**B**) cells were incubated with a 10 µM concentration of GRNLY (upper panel) or of SM3GRNLY (black bars) during the times indicated. After the incubations, cell death was determined by detecting PS exposure by Annexin-V-FITC (**A**) or Annexin V-DY63 (**B**) labeling and analysis of cell size by FSC. Results are the mean ± SD of three independent experiments; ** *p* < 0.01.

In this work, we have studied the mechanism of cell death induced in tumor cells by the anti-Tn immunotoxin SM3GRNLY. For that, we first tested SM3GRNLY on the pancreatic adenocarcinoma cell line MIA PACA-2 and on its derivative in which Bax and Bak were silenced, MIA PACA-2-KO. While the cytotoxic effect of GRNLY was highly compromised in MIA PACA-2-KO cells, that of SM3GRNLY was not significantaly affected (see Figure 7A). To confirm these results, we also performed a dose-response test of SM3GRNY and GRNLY on Jurkat cells in which pro-apoptotic Bak was deleted by small hairpin technology, generating the Jurkat-shBak cell line. Jurkat cells do not express Bax, so Jurkat-shBak cells are unable to activate the mitochondrial apoptotic pathway [32]. A comparative time course was performed with a 10 µM concentration of each protein in Jurkat cells, Jurkat cells transfected with the empty vector used to generate the Bak-deficient subline, Jurkat–pLVTHM cells, or Jurkat-shBak cells. As shown in Figure 7B, the cytoxicity of GRNLY is similar on Jurkat and on Jurkat-pLVTHM cells and this toxicity is almost totally abolished in Jurkat-shBak cells. In the case of SM3GRNLY, its toxicity was higher than that of GRNLY on Jurkat and on Jurkat-pLVTHM cells and, although lower, it was not eliminated on Jurkat-shBak cells, suggesting the activation of a cell death mechanism independent of the mitochondrial apoptotic pathway.

**Figure 8:**
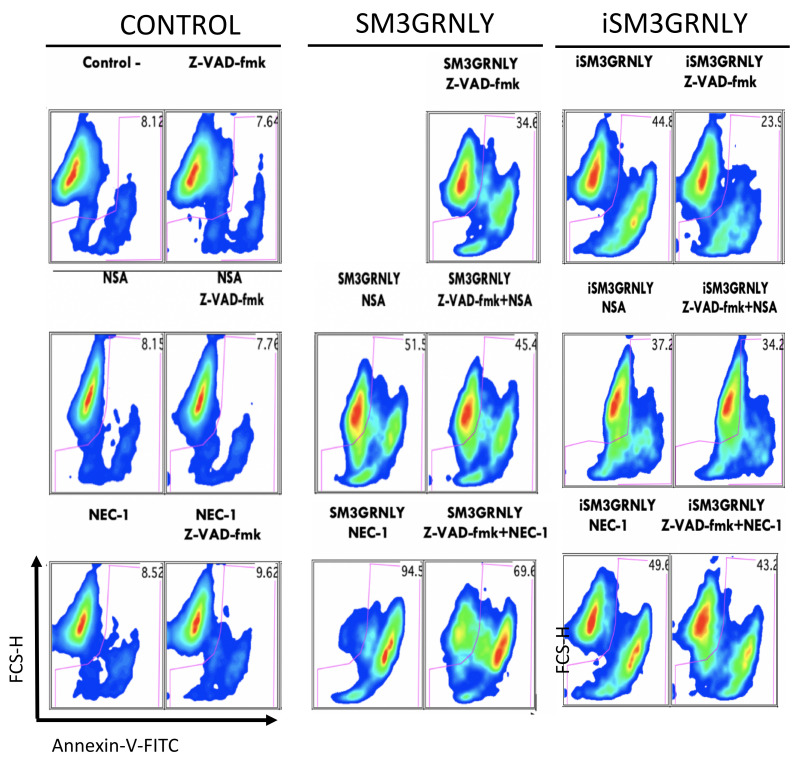
Study of the induction of death in JURKAT cells. Cells were preincubated for 1h in the presence or absence of 100 µM Z-VAD-fmk, 30 μM NEC-1 or 1 µM NSA alone or in combination. Subsequently, they were incubated with 10 µM of SM3GRNLY for 24 h and analyzed by flow cytometry by labeling with Annexin-V-FITC combined with the analysis of cell size in FSC-H. Representative images from two independent experiments are shown.

Another strategy to study the mechanism of death induction by SM3GRNLY was the use of inhibitory molecules of known death pathways. The general caspase inhibitor Z-VAD-fmk significantly, although only partially, prevented death induced by SM3GRNLY (from 98% to 34%), while the RIP1 inhibitor, necrostatin 1 (NEC-1) had no effect either alone or in combination with Z-VAD-fmk (Figure 8). The MKLK inhibitor N-sulfonamide (NSA) partially prevented the death induced by SM3GRNLY (from 98% to 51%) and this inhibition increased when combined with Z-VAD-fmk up to 45%. However, inhibition was not complete in any case. These results suggest that, while GRNLY exerts tumor cell death following the classical caspase dependent, mitochondrial apoptotic pathway, SM3GRNLY-induced cell death implicates both apoptotic and necroptotic pathways, together with a rather necrotic component. Similar results were obtained for iSM3GRNLY (see Supplementary Figure S2).

### 3.6. In Vivo Demonstration of Immunotoxin Targeting after Systemic Injection

The main objective of granulysin-based immunotoxin design was to demonstrate that they are able to target the toxic moiety towards Tn–expressing tumors after systemic injection in vivo.

For that, we first selected the Tn-expressing cell line for conducting in vivo assays attending to three parameters:

- tumor development in a reasonable time after injection in athymic mice
- high immunotoxin binding
- increase in cytotoxicity of the immunotoxins as compared with GRNLY

For example, although the immunotoxins bound with high intensity to Jurkat and Panc-1 cells ([22] and Figure 4), indicative of a high MUC1-Tn antigen expression, these cells did not generate tumors in athymic mice after s.c. injection of 10 × 10^6^ cells for more than three months, so these cell lines were discarded for in vivo experiments. The cell line that best attained the three parameters was the pancreatic adenocarcinoma Capan-2. This cell line generated detectable tumors in athymic mice between 10 and 20 days after injection of 5 × 10^6^ cells in Matrigel; the binding of SM3GRNLY, iSM3GRNLY and especially of AR20.5GRNLY to this cell line is maximal (Figure 4); and while the IC50 for GRNLY was 10 µM, it was reduced to 5 µM for SM3GRNLY and iSM3GRNLY (Figure 5) and to 2.5 µM for AR20.5GRNLY (Figure 6). Hence, Capan-2 was selected to perform the in vivo assays of the immunotoxins compared with GRNLY. This is especially interesting, given the bad prognosis of pancreatic adenocarcinoma and the lack of efficient treatments.

**Figure 9:**
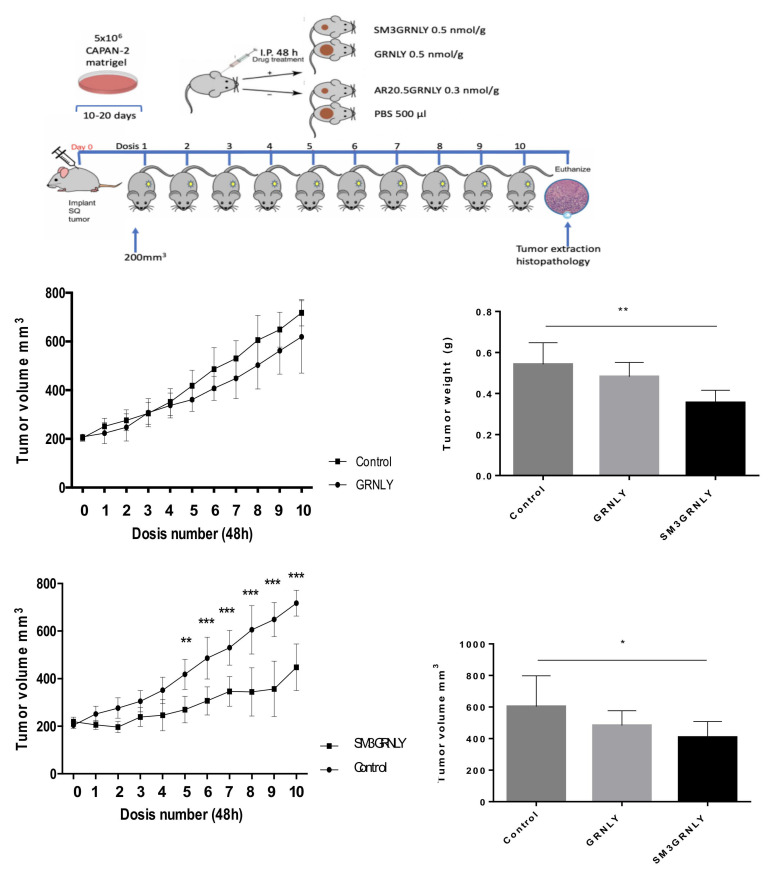
Systemic treatment of nude mice xenografted with CAPAN-2 cells. The mice in each group received intraperitoneal injections of GRNLY or of SM3GRNLY, as indicated, every 48 h for 10 occasions and two days after the last dose, the animals were sacrificed. Mice in the control group received PBS injections with the same schedule. Line graphics show the mean ± SD of tumor volume as a function of time in the control group and in GRNLY-treated mice (upper graphic) or in the control group and in SM3GRNLY-treated mice (lower graphic), Bar graphs correspond to the mean ± SD of the weights (upper graphic) or volumes (lower graphic) of surgically excised tumors from sacrificed mice in each experimental group, as indicated. * *p* < 0.05; ** *p* < 0.01; *** *p* < 0.001.

The protocol followed in these experiments is shown in the upper panel of Figure 9 and described in the Materials and Methods section. As shown in the middle upper panel of Figure 9, the inhibition of tumor growth by systemic injection of GRNLY was not significant at any time point. However, SM3GRNLY was effective in inhibiting the growth of Capan-2-derived tumors, with the effect being statistically significant from the fifth injection of the treatment. The inhibition of tumor growth by SM3GRNLY at the time of sacrifice was 42% (lower middle panels of Figure 9). When analyzing the tumor weights and volumes of resected tumors (bar graphics), the SM3GRNLY inhibition of tumor growth was also statistically significant and accounted for 40 and 33% of inhibition, respectively.

Regarding AR20.5GRNLY, and due to the above-described problems of production, we could only produce recombinant pure protein to treat one mouse, so the results do not have statistical significance. As shown in Supplementary Figure S3, AR20.5GRNLY substantially reduced tumor growth in this mouse, arriving to 60% of tumor growth inhibition at the time of sacrifice.

**Figure 10:**
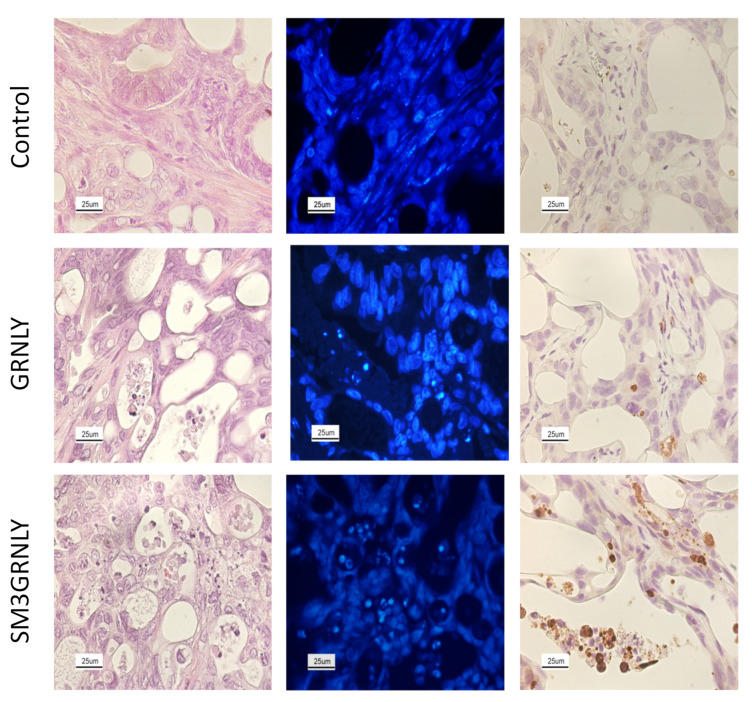
Hematoxylin-Eosin staining (**left**), DAPI nuclear staining (**middle**) and activated caspase-3 immunohistochemistry (**right**) of tumors derived from CAPAN-2 xenografts. Representative images of histological sections of tumors from mice treated with PBS (**first** row), GRNLY (**second** row) or SM3GRNLY (**third** row). Image magnification was used at 400×.

After tumor resection, we performed histological analysis of tumors obtained from treated or untreated mice and representative images are shown in Figure 10. As shown in the left panels of Figure 10, the hematoxilin/eosin staining of tumors obtained from control mice showed high cellularity and the presence of acinar structures, typical of this type of glandular tumor. However, in the sections of tumors obtained from mice treated with SM3GRNLY, cellularity is reduced and the glandular acinar spaces are disorganized.

Nuclear staining in tumor sections was performed using the DAPI fluorescent molecule and representative images are shown in the middle panels of Figure 10. Tumors obtained from mice treated with SM3GRNLY showed chromatin condensation and nuclear fragmentation, typical of apoptotic cell death, a pattern that was absent in the tumors of untreated mice and scarce in the tumors of mice treated with GRNLY. 20 images were analyzed per experimental group, typically observing no more than 3 nuclei fragmented or with condensed chromatin per image in the control group, a maximum of 6 in the images of the GRNLY group and at least 15 in the images of the SM3GRNLY-treated group.

To further ascertain apoptosis induction in tumors from treated mice, we determined activated caspase-3 in the tumor tissue by immunohistochemistry. While no active caspase-3 was detected in tumors from control mice, and very scarce staining was obtained in tumors from mice treated with GRNLY, brown stained cells was very apparent in tumors obtained from mice treated with SM3GRNLY (Figure 10). 20 images were analyzed per experimental group, typically observing no more than 1 cell positive for the staining per image in the control group, a maximum of 5 in the images of the GRNLY group and at least 16 in the images of the SM3GRNLY-treated group.

Similar results were obtained in the indicated histological studies in the tumor obtained from the AR20-5GRNLY-treated mouse (Supplementary Figure S4).

## 4. Discussion

The antibacterial and antiparasitic function of 9 kDa GRNLY has long been known, a property that has been confirmed and expanded in recent studies [33,34,35]. Our group has shown that recombinant GRNLY is capable of killing tumor cells in vitro through the mitochondrial apoptotic pathway [17,18,27]. This antitumor activity of recombinant GRNLY was also demonstrated in vivo, initially by intratumoral injection in several tumors types: mammary carcinoma, multiple myeloma and melanoma [17,18]. There are abundant data that relate granulysin expression to an active antitumor immune response and to a good prognosis [36,37,38,39]. Although mice do not express a granulysin homologue, the Alan Krensky group generated transgenic mice that expressed human granulysin and showed an increased resistance to tumor development [40].

However, systemic treatment of tumors would require improving the directionality of the molecule. This can be achieved by the conjugation of an antibody or antibody fragment directed against a tumor molecule with GRNLY, generating a so-called fourth-generation immunotoxin [16]. Our team designed three immunotoxins that conjugated GRNLY with scFv, one directed against CEA (Carcino-embryonic antigen) [21], and two against the Tn antigen [22]. CEA or CEACAM5 is a tumor antigen expressed preferentially by colorectal or gastric tumors [41]. The Tn antigen is an aberrant glycosylation of the surface protein MUC-1 and is expressed by a wide variety of tumors [2,3]. The systemic administration of the anti-CEA immunotoxin, called MFE23GRNLY, has shown directionality against tumors positive for CEA expression and cytotoxic capacity superior to GRNLY in a murine model [21] constituting the proof of concept of this new anti-tumor treatment.

The efficiency of electroporation by PEF showed that a high percentage of the two anti-Tn used in this work, SM3GRNLY and AR20.5GRNLY, was retained inside yeasts, which was not the case with the GRNLY or MFE23GRNLY producing strains, in which most of the protein is secreted by yeasts to the supernatant (Raquel Ibáñez, personal communication). The use of the PEF technique has allowed to perform the in vivo experiments shown, which need higher amounts of the recombinant proteins [22].

According to the SRP assays, immunotoxins retained the ability to recognize and bind its antigen with a similar affinity that was described previously for the scFv fragments alone. The ability to bind to the Tn antigen expressed on the surface of living tumor cells was also demonstrated in cell lines in which the expression of the Tn antigen had been described: pancreatic adenocarcinomas PANC-1, CAPAN-2 [42], MCF-7 breast adenocarcinoma [43], A549 lung adenocarcinoma [44] and Jurkat acute lymphocytic leukemia [29], using MDA-MB-231 mammary adenocarcinoma as a negative control [26]. In general, this recognition was of greater intensity, and in a greater number of cell lines, with the immunotoxin AR20.5GRNLY with respect to SM3GRNLY and iSM3GRNLY.

The binding of the anti-Tn immunotoxins used in this work to its antigen, purified or expressed on the surface of tumor cells was correlated with a higher cytotoxicity on different cell lines positive for the expression of Tn compared with recombinant GRNLY alone. We also demonstrate that these immunotoxins exert cytotoxicity on tumor cells by combining several cell death mechanisms: apoptosis, necroptosis and necrosis. More importantly, these immunotoxins demonstrated directionality of the treatment after systemic injection, reducing the tumor volume of xenotransplants of CAPAN-2 human pancreatic adenocarcinoma in athymic mice, whereas recombinant GRNLY alone was not effective. Tumor size reduction was associated with induction of apoptosis in the tumor tissue, demonstrated by nuclear morphology analysis and in-tissue caspase-3 activation. One limitation of the proposed immunotoxins would be their binding to soluble MUC1 fragments in serum, neutralizing their anti-tumor activity. The release of such fragments have been described, especially in breast cancer patients, and termed the CA15-3 antigen. However, CA15-3 is elevated in only a 10% of breast cancer patients and few studies have explored its association with patient prognosis [45].

The mechanism by which granulysin results cytotoxic against tumor cells is mediated by the interaction of its positive charges with negatively charged membrane phospholipids [46]. This interaction is dependent on the presence of a net negative charge of the tumor cell membrane and is reduced when the cholesterol/phospholipid ratio increases. This explains why granulysin is especially active on lipid membranes of microorganisms devoid of cholesterol, such as bacterial membranes, and less active on eukaryotic lipid membranes [47]. However, the cholesterol/phospholipid ratio of tumor cells is highly reduced as compared with that of their healthy counterparts [48,49], explaining the tumor selectivity of granulysin [30,50]. Although granulysin, contrary to perforin, is not able to induce the formation of pores in the membrane, its interaction with phospholipids induces alterations of the membrane structure enough to alter the cellular ionic equilibrium [46], resulting in a net increase the cytoplasmic Ca^2+^ concentration [19,51]. This increase in the cytoplasmic Ca^2+^ concentration causes the generation of mitochondrial ROS that leads to the loss of mitochondrial membrane potential and release of the apoptogenic molecules cytochrome c and AIF from mitochondria, initiating the mitochondrial apoptotic pathway and caspase-independent cell death, respectively [17,46,47]. When granulysin is acting in concert with perforin, the mechanism of cell death induction is rather dependent of ER stress and seems to activate a different set of executor caspases [52].

One of the main problems of immunotoxins that include bacterial or plant toxins, such as the approved moxetumomab pasudotox [15] is the high immunogenicity of the toxin moiety [53]. In patients with hematological malignancies, the formation of neutralizing antibodies against the toxins is low, probably due to the immunosuppressed situation of these patients. However, in patients with solid tumors, the rate of antibody formation is higher. Mutagenic de-immunization of those toxins has been proposed and new versions of PE-based immunotoxins have been generated to alter epitopes recognized by neutralizing antibodies or by T cells [54,55]. Another approach to solve this problem has been the design of the so-called 4th generation immunotoxins, which include toxic moieties of human origin. Our granulysin-based immunotoxins would be included in this group, that would be devoid of this immunogenicity [16].

In addition, the membrane-based activity of recombinant granulysin or of granulysin-based immunotoxins constitutes a new immunotoxin mechanism of action, that is non-dependent on internalization and release from the endosome, one of the major caveats of immunotoxin use, including granzyme B-based 4th generation immunotoxins [56].

Remarkably, the antitumor activity of granulysin was associated with a massive NK cell infiltration, suggesting a possible immunogenic effect of granulysin-induced tumor cell death [17,18]. In addition, no side effects of this type of treatment were detected in those in vivo experiments [17].

Together with our previous study using the anti-CEA immunotoxin MFE23GRNLY [21], the present work opens the door to the use of granulysin-based immunotoxins for cancer treatment, expanding also its application to a broader spectrum of cancer types in the case of immunotoxins directed against the Tn antigen.

## 5. Conclusions

We have generated two granulysin (GRNLY) based immunotoxins, SM3GRNLY and AR20.5GRNLY, directed against the MUC-1-Tn antigen. We showed their affinity for their antigen in vitro and also on the surface of a panel of tumor target cells, correlating with their increased cytotoxicity if compared with GRLY alone. We demonstrated in vivo tumor targeting by the immunotoxins after systemic injection in a xenograft model of the human pancreatic adenocarcinoma CAPAN-2 in athymic mice. Overall, our results indicate that the immunotoxins developed were able to increase the antitumor potential of granulysin and to improve its in vivo targeting towards MUC-1-Tn^+^ tumors.

## Data Availability

The data presented in this study are available on request from the corresponding author.

## Supplementary Materials

The following supporting information can be downloaded at: https://www.mdpi.com/article/10.3390/biomedicines10061223/s1, Table S1: Yield of the production of the recombinant proteins using different purification techniques; Figure S1: Binding and cytotoxicity of the SM3GRNLY or the AR20.5GRNLY immunotoxins to PBMC obtained from the blood of healthy donors; Figure S2: Study of the induction of death by iSM3GRNLY on Jurkat cells; Figure S3: Systemic treatment of nude mice xenografted with CAPAN-2 cells with GRNLY or AR20.5GRNLY; Figure S4: Histological studies of tumors derived from CAPAN-2 xenografts obtained from mice treated with GRNLY or AR20.5GRNLY
