# Supplementary material for: Preclinical Studies of Granulysin-Based Anti-MUC1-Tn Immunotoxins as a New Antitumoral Treatment
Source: Biomedicines. 2022 May 24;10(6):1223. doi: 10.3390/biomedicines10061223 (PMC9219680; doi:10.3390/biomedicines10061223)
Supplement: Supplementary file 1 [file biomedicines-10-01223-s001.zip › biomedicines-1724154-supplementary.pdf]

Table S1. Yield of the production of the recombinant proteins using different purification techniques. Data is shown as the mean in mg or nmol of protein produced per liter of *P.pastoris* culture.

Protein	Source	Yield (mg/L)	Molar Yield (nmol/L)
GRNLY	Extracellular	5.040	458
SM3GRNLY	Extracellular	2.115	45
iSM3GRNLY	Intracellular	8.260	176
AR20.5GRNLY	Extracellular	0.555	12
iAR20.5GRNLY*	Intracellular	12.891	292

* Included a major impurity.

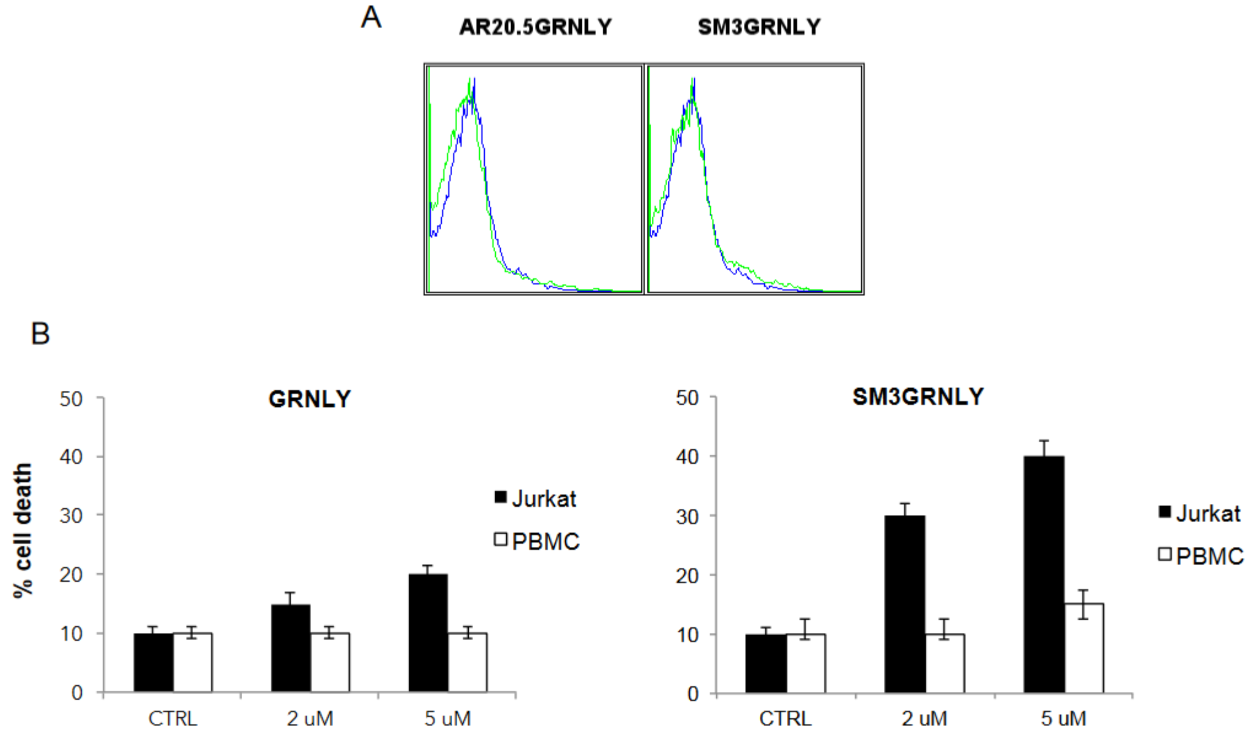

Figure S1. (**A**) Binding of the SM3GRNLY or the AR20.5GRNLY immunotoxins to PBMC obtained from the blood of healthy donors. Green histograms correspond to cells incubated with anti-His-tag antibody and FITC-conjugated goat anti-mouse IgG antibody in the absence of the immunotoxin and blue histograms correspond to cells sequentially incubated at 4 °C with the immunotoxins and the mentioned antibodies. (**B**), cytotoxicity of GRNLY or SM3GRNLY on PBMC and Jurkat cells. Jukat (black bars) or PBMC (white bars) were incubated with the indicated concentrations of the recombinant proteins GRNLY (left panel) or SM3GRNLY (right panel) for 24 h. Cell death was determined by detection of PS translocation by Annexin-V-FITC labeling combined with size analysis. Results are the mean ± SD of two different experiments performed in triplicate.

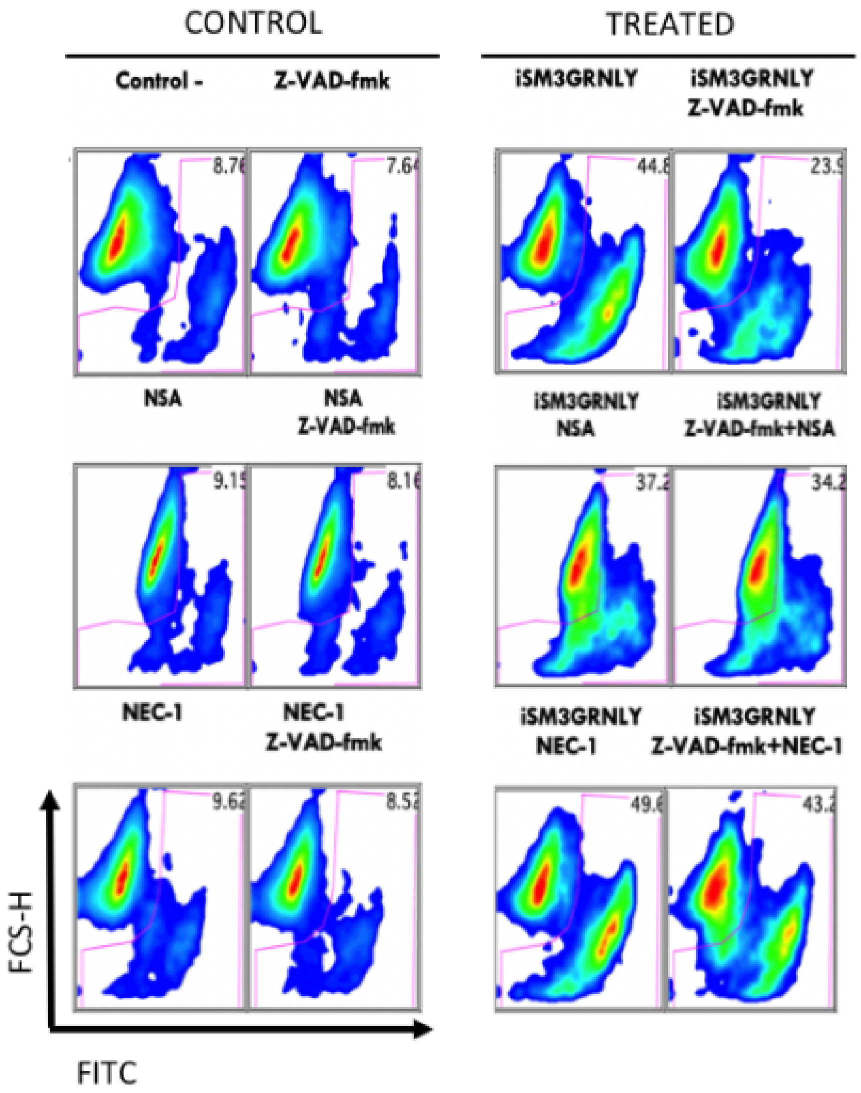

Figure S2. Study of the induction of death by iSM3GRNLY on Jurkat cells. Cells were preincubated for 1 h in the presence or absence of 100 µM Z-VAD-fmk, 30 µM NEC-1 or 1 µM NSA alone or in combination. Subsequently, it was incubated with 10 µM of iSM3GRNLY for 24 h and analyzed by flow cytometry by labeling with Annexin-V-FITC combined with the analysis of cell size in FSC-H. Representative image from two independent trials.

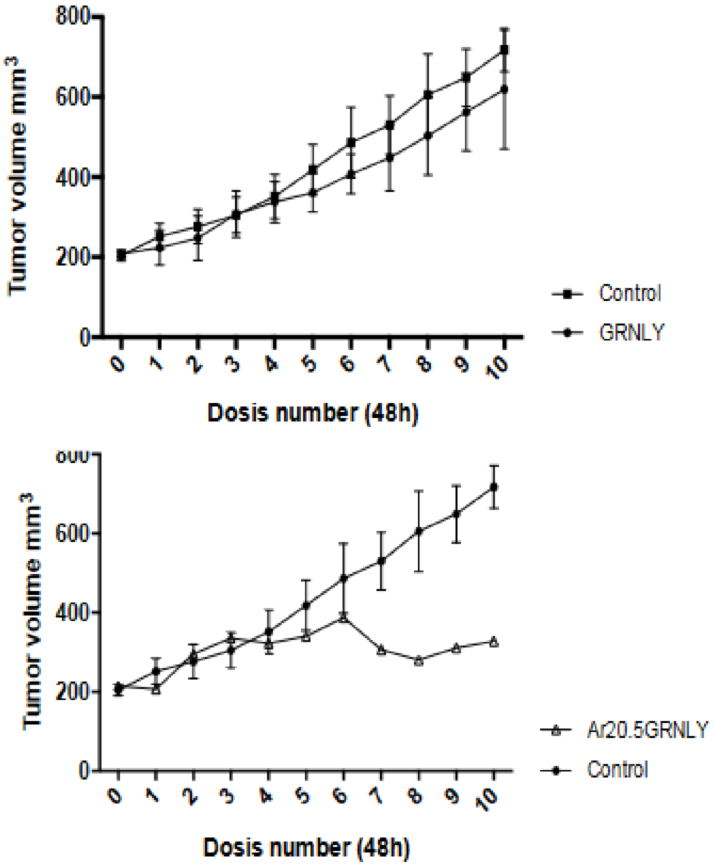

Figure S3. Systemic treatment of nude mice xenografted with CAPAN-2 cells with GRNLY or AR20.5GRNLY. The mice in each group received intraperitoneal injections of GRNLY or of AR20.5GRNLY, as indicated, every 48h for 10 occasions and two days after the last dose, the animals were sacrificed. Mice in the control group received PBS injections with the same schedule. Graphics show the mean ± SD of tumor volume as a function of time in the control group and in GRNLY-treated mice (upper graphic) or in the control group and in a single mouse treated with AR20.5GRNLY (lower graphic).

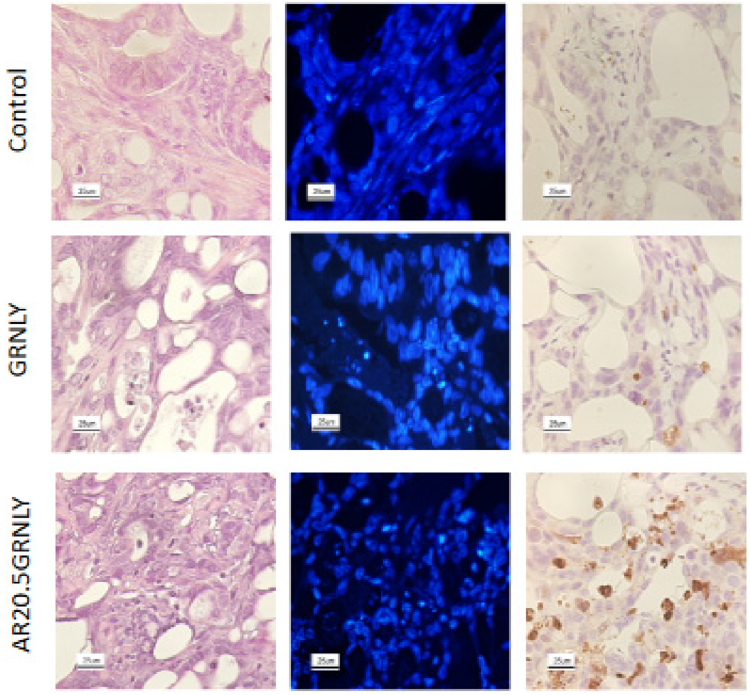

Figure S4. Histological studies of tumors derived from CAPAN-2 xenografts obtained from mice treated with GRNLY or AR20.5GRNLY. Hematoxylin-Eosin staining (left), DAPI nuclear staining (middle) and activated caspase-3 immunohistochemistry (right) of tumors derived from CAPAN-2 xenografts obtained from control mice or from mice treated with GRNLY or AR20.5GRNLY, as indicated. Image magnification was used at 400×.
